# Senescence under appraisal: hopes and challenges revisited

**DOI:** 10.1007/s00018-020-03746-x

**Published:** 2021-01-13

**Authors:** Camilla S. A. Davan-Wetton, Emanuela Pessolano, Mauro Perretti, Trinidad Montero-Melendez

**Affiliations:** 1 The William Harvey Research Institute, Barts and The London School of Medicine, Queen Mary University of London, Charterhouse Square, London, EC1M 6BQ UK; 2 Department of Pharmacy, University of Salerno, via Giovanni Paolo II 132, 84084 Fisciano, Italy; 3 Centre for Inflammation and Therapeutic Innovation, Queen Mary University of London, London, EC1M 6BQ UK

**Keywords:** Senescence, Tissue repair, Ageing, Cancer, Senolytics, Resolution of inflammation

## Abstract

In recent years, cellular senescence has become the focus of attention in multiple areas of biomedical research. Typically defined as an irreversible cell cycle arrest accompanied by increased cellular growth, metabolic activity and by a characteristic messaging secretome, cellular senescence can impact on multiple physiological and pathological processes such as wound healing, fibrosis, cancer and ageing. These unjustly called ‘zombie cells’ are indeed a rich source of opportunities for innovative therapeutic development. In this review, we collate the current understanding of the process of cellular senescence and its two-faced nature, i.e. beneficial/detrimental, and reason this duality is linked to contextual aspects. We propose the senescence programme as an endogenous pro-resolving mechanism that may lead to sustained inflammation and damage when dysregulated or when senescent cells are not cleared efficiently. This pro-resolving model reconciles the paradoxical two faces of senescence by emphasising that it is the unsuccessful completion of the programme, and not senescence itself, what leads to pathology. Thus, pro-senescence therapies under the right context, may favour inflammation resolution. We also review the evidence for the multiple therapeutic approaches under development based on senescence, including its induction, prevention, clearance and the use of senolytic and senomorphic drugs. In particular, we highlight the importance of the immune system in the favourable outcome of senescence and the implications of an inefficient immune surveillance in completion of the senescent cycle. Finally, we identify and discuss a number of challenges and existing gaps to encourage and stimulate further research in this exciting and unravelled field, with the hope of promoting and accelerating the clinical success of senescence-based therapies.

## Introduction, definitions and history

Senescence, /sɪˈnɛs(ə)ns/, *noun*—the condition or process of deterioration with age

Dating back to the seventeenth century, the term senescence was used to refer to the general and gradual accumulation of changes over time leading to the decline of organismal function, essentially aging. In this context, this process is now referred to as *organismal senescence*. Senescence also applies to plants, appreciable during autumn when chlorophyll is recycled revealing the bright red leaf colour of carotenoids, and to unicellular organisms like bacteria when they exhibit decreased growth rate [1]. However, the type of senescence, subject of this review, that has recently captured the attention of the scientific community, pharmaceutical industry and even the general public is known as *cellular senescence*, typically defined as the irreversible or stable cell cycle arrest even in the presence of mitogenic stimuli [2, 3]. This rather simple definition encompasses a very complex and highly heterogeneous phenomenon, in which the stable cell cycle arrest can be driven by multiple mechanisms and associated with diverse phenotypes and functions. Together with loss of proliferative potential, a key feature that distinguishes senescence from other arrested cellular states, such as quiescence or terminal differentiation, is the acquisition of the ‘senescence-associated secretory phenotype’ (SASP) [4], which has strong implications on the outcome of senescence and in the development of therapeutic interventions.

The discovery of cellular senescence in 1961 by Hayflick and Moorhead [5] represented a ground-breaking finding as it was the first demonstration that normal cells are not immortal, as believed at the time, and provided the basis for a scientific explanation of the evolutionary theory of ageing initiated by Peter B. Medawar in 1952. In his lecture ‘An unresolved Problem of Biology’ delivered at University College London, he stated that the force of natural selection declines with age as the probability of an organism to exist declines (e.g. due to diseases, accidents, predators) in parallel to its reproductive value, a concept known as *selection shadow*. Hence, gene variants that are beneficial in early life are favoured over genes beneficial late in life, in perfect agreement with Darwinian theories. George C. Williams built on this model and introduced in 1957 the concept of *antagonistic pleiotropy* [6], based on genes having multiple functions: harmful late-acting genes can remain and accumulate in the population if they have a beneficial effect early in life. Hence, the biological and inevitable mortality of cells discovered by Hayflick and Moorhead fitted with Medawar and Williams model of ageing: cellular senescence is evolutionary conserved, because it confers survival advantage early in life, even if, past reproduction age, manifests deleterious effects. The cost–benefit in evolutionary terms is favourable.

Forty years on, our view of cellular senescence has been expanded, shaken, refined and now apparently settled on the consensus that the actual process of senescence is necessary and desirable to avoid propagation of cellular damage, but it is the failure of timely clearing of these cells from tissues that leads to persistent damage, a process where the (aged) immune system might play a major role, as will be discussed later. Worth mentioning at this point is the concept of *immunosenescence* which broadly speaking refers to the general and progressive decline of immune function during ageing. Senescence in T cells refers to the loss of proliferative potential generally resulting from excessive telomere erosion and associated with increased cytokine release [7]. However, it is not clear what immunosenescence refers to in terminally differentiated, hence non-proliferative, immune cells like, for example, neutrophils. Pears and apples are being mixed under the same label. Beyond a perhaps irrelevant discrepancy on definitions, an immediate consequence is that markers normally used to identify and study cellular senescence may be unsuitable to study immunosenescence, like proliferation arrest markers. Even in T cells, where senescence fits with the accepted definition of *cellular* senescence, it is not clear whether the typical markers p16 or β-galactosidase are involved [7]. Hence, a consensus of what constitutes immunosenescence and specific definitions applicable to proliferative and terminally differentiated immune cells are still needed.

In this review, we present senescence in the context of the resolution of inflammation and propose that a failure of this endogenous protective mechanism leads to persistent damage and inflammation. We also highlight the heterogeneity of senescence, how this diversity impacts research and opportunities and we bring together different views, contradictory only in appearance, on the role of senescence in health and disease and why consideration of ‘context’ is determining. In addition, we discuss the influence of the immune system in the successful completion of the senescence programme and how senescence can be harnessed therapeutically from multiple angles. Finally, we raise existing knowledge gaps and current challenges that need to be addressed to advance the therapeutic exploitation of senescence, hoping to stimulate and encourage the field to transform patient care based on strategies targeting senescence in multiple therapeutic areas such as ageing, tissue repair and cancer.

## The endogenous defensive role of cellular senescence

### General pathways and mission of senescence

Cellular senescence is a state of permanent cell cycle arrest induced in proliferating cells by multiple stressors and conditions. The most common mechanism involves the activation by p53 of cyclin-dependent kinase (CDK) inhibitors, such as p16 (*CDKN2A*), p15 (*CDKN2B*), p21 (*CDKN1A*) or p27 (*CDKN1B*). These inhibitors block the actions of CDK/cyclins complexes, preventing the phosphorylation of the retinoblastoma protein, Rb [3]. Hypophosphorylated Rb remains bound to the transcription factor E2F, preventing the transition of the cell cycle from G_1_ to S phase. This loss of proliferative potential occurs even in the presence of serum or growth factors, a key difference from quiescence, where cells, whilst non-proliferative, retain the *potential* to proliferate again when mitogenic stimuli become available [8]. Quiescence is an *exit* from the cell cycle into G_0_, while senescence is an *arrest* in G_1_. This implies another key distinctive feature of senescent cells: although proliferation is halted, growth is still ongoing, reflected in the increase in cell size, lysosomes and metabolic activity, all hallmarks of cellular senescence [2, 9, 10]. Quiescence is a stand-by mode, while senescence is a flight forward.

The increased activity of senescent cells results in a characteristic messaging secretome, the SASP, that confers new functions to the senescent cells. This is an important difference with apoptosis. Although both processes seem to share the mission of eliminating damaged cells and limit the propagation of the damage to daughter cells, it is not completely clear what determines one fate or another. Some redundancy exists, as there is evidence that one can compensate, to some extent, for the absence of the other [11]. Apoptosis is per se a cell death mechanism, but senescent cells remain alive for long periods of time [12], as if they could still offer a last service to the host before ultimately being eliminated. This last service is offered by the SASP [13, 14], a combination of factors that initiate the tissue repair programme and attract the immune system to induce their own clearance. The SASP also amplifies the senescence process by inducing secondary senescence to neighbouring cells either via soluble factors [15, 16] or via extracellular vesicles [17] and contributes to their increased survival. Moreover, the SASP can also promote a pro-regenerative response by favouring cell plasticity and stem cell activity [18] and induce vascular remodelling [19].

In summary, both arms of senescence, proliferation arrest and secretome, play specific homeostatic and defensive roles in physiological and pathological situations. This is discussed next.

### Cancer, tissue repair and development

The first type of senescence discovered by Hayflick and Moorhead [5], now referred to replicative senescence, is caused by the progressive shortening of telomeres after a limited number of cell divisions resulting in the activation of the DNA damage response (DDR) once a critical telomere length has been reached. This response leads to senescence, preventing proliferation of aberrant cells and inhibiting tumour formation [20]. Similarly, the DDR can be triggered by the acquisition of activating or inactivating mutations in oncogenes or tumour suppressor genes, respectively, leading to the activation of mainly p53 and p16 or p21, initiating the senescence programme [3]. This oncogene-induced senescence was first discovered for the gene *HRAS* [21] and later on for other genes such as *PTEN*, *MYC*, *BRAF*, *TP53*, *RAC1* and many others [22], and represents a potent endogenous anti-cancer mechanism acting at very early stages of tumorigenesis. Melanocytic nevi on skin surface are a visible example of oncogene-induced senescence, in which *BRAF* is mutated in ~ 80% of the cases [23] typically acquiring the activating mutation *BRAF*^V600E^. This illustrates the essential protective role of senescence under the constant environmental oncogenic pressure exerted by UV radiation. Indeed, germline mutations in *CDKN2A,* permitting senescence bypass, greatly increase melanoma susceptibility [24]. Inactivating mutations in other pro-senescence mediators like *CDKN1A*, associated with bladder cancer [25], or *RB1*, linked to retinoblastoma, provide further evidence of the anti-tumour role of senescence. In mice, mutations in *CDKN2A* gene, affecting either or both encoded proteins p16 and p19, or in *CDKN1A,* result in increased tumour development susceptibility [26, 27].

The senescence programme also orchestrates tissue remodelling associated with development as well as wound healing. Senescence occurs in the embryo in multiple structures like the endolymphatic sac, the inner ear, the apical ectodermal ridge and neural tube, and although some redundancy exists with apoptosis, impaired senescence in certain structures can result in morphological defects [3, 11, 28]. Senescence is also involved in the regulation of placental formation and function, and a deficient senescence programme has been associated with pregnancy complications like intrauterine growth restriction [29].

The role of senescence in tissue remodelling in the adult is also well established. Importantly, the SASP released by senescent cells influences the microenvironment orchestrating the process of wound healing. Proliferating myofibroblasts at early stages of wound healing, are driven into senescence by matricellular proteins like CCN1 during the remodelling phase [30]. Much evidence suggests that senescent cells get locked in a pro-remodelling state characterized by a reduction in collagen deposition and increase in the expression of remodelling enzymes like matrix metalloproteases. This characteristic pro-remodelling signature has been shown in transcriptomic analysis of dermal senescent fibroblasts [31], in mouse models of cutaneous wound healing [30, 32], in hepatic stellate cells [33] and in synovial fibroblasts from rheumatoid arthritis [34]. Interestingly, these examples represent very distinct types of senescence, including replicative, chemically induced and MC_1_ agonist-mediated senescence, suggesting that the pro-remodelling phenotype may be a common feature of senescence. Furthermore, repair can be impaired by the active elimination of senescent cells, using either the p16-3MR mouse model [32] or administration of senolytics [34]. Physiological pre-programmed cellular senescence in the adult also occurs during thymic involution [35] and megakaryocyte maturation [36].

### Senescence as a pro-resolving mechanism

The termination of the inflammatory response is achieved by a number of endogenous mediators that promote the active and safe completion of the inflammatory response leading to restoration of homeostasis. The realization of the *active*, rather than passive nature of this process, gave birth to a new field of research, the resolution of inflammation [37], and to a new strategy to target inflammatory conditions, resolution pharmacology [38], based on promoting and reinforcing those endogenous pathways and mediators. Another important insight was the realisation that chronic inflammation can derive from the actual failure of these endogenous pathways. Conditions such as rheumatoid arthritis [39], atherosclerosis [40] and inflammatory bowel disease [41] are now seen as a failure of resolution and among the pro-resolving pathways and mediators under pre- and clinical investigation are melanocortins [42], formyl-peptide receptors [43] and lipid resolvins [44].

A common feature of non-resolving inflammation is the dysregulated persistence of certain cells after the inflammatory insult has been neutralized, cells that keep dancing when the music has already stopped. Persistence of myofibroblasts that refuse to die by evading apoptosis impairs wound resolution by promoting fibrosis [45]. Persistence of activated neutrophils with increased lifespan or impaired clearance can delay resolution by causing excessive tissue damage [46]. Similarly, *persistence* of senescent cells prevents resolution by the influence of damaging SASP. Persistent myofibroblasts can be targeted with pro-apoptotic drugs like ABT-263 [47], similar to persistent neutrophils which can be eliminated with CDK inhibitors like R-roscovitine [46]. Hence, *persistent* senescent cells can similarly be targeted for elimination to induce resolution.

**Fig. 1:**
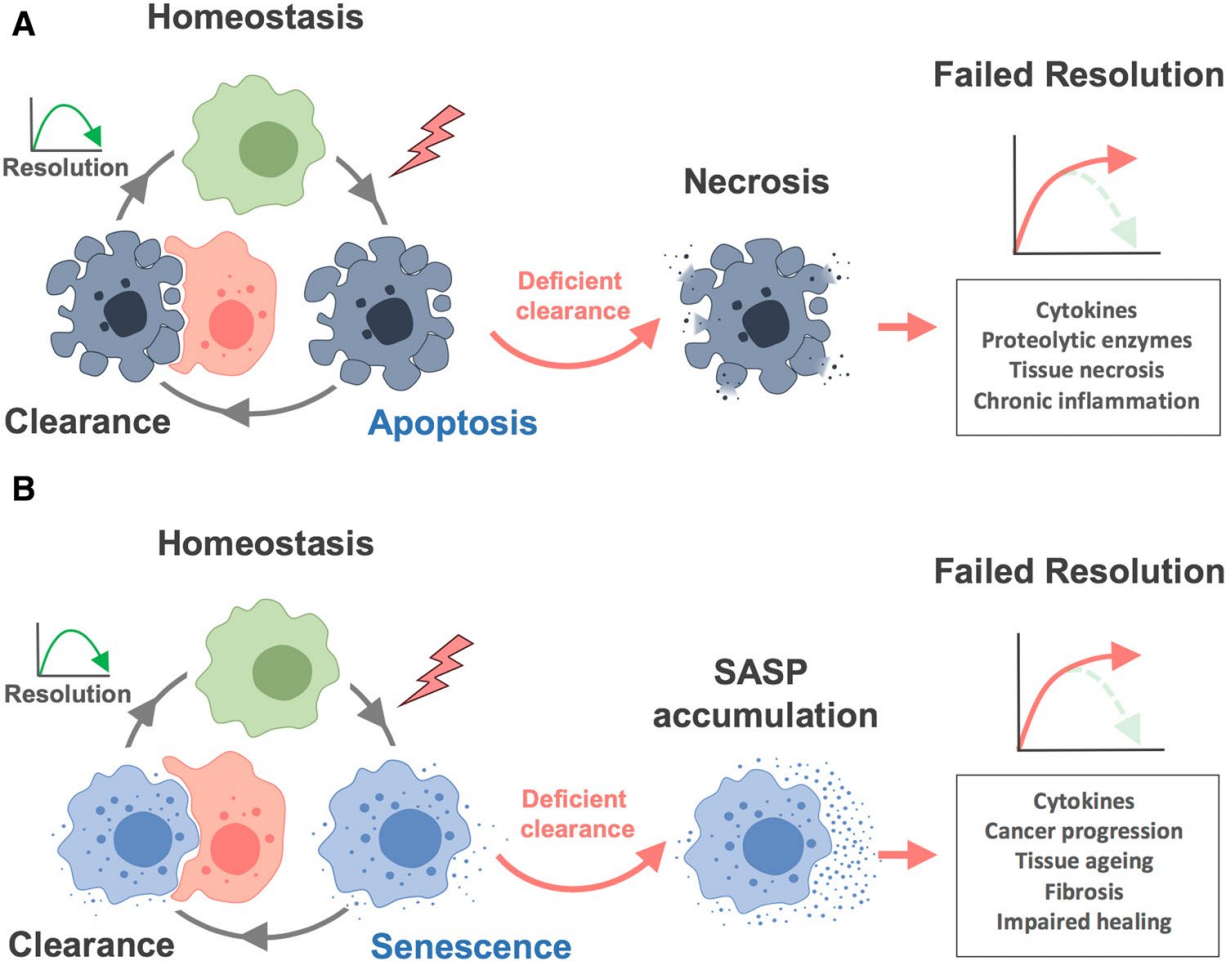
Cell clearance and the resolution of inflammation. **a** The resolution of inflammation requires the efficient and timely clearance of apoptotic cells from tissues by efferocytosis. A defective clearance may lead to secondary necrosis further promoting inflammation. **b** Similarly, the clearance of senescent cells, efficient and timely, is also required to complete the senescence programme successfully. An impaired clearance will lead to persistent action of SASP components leading to a failure of resolution

**Fig. 2:**
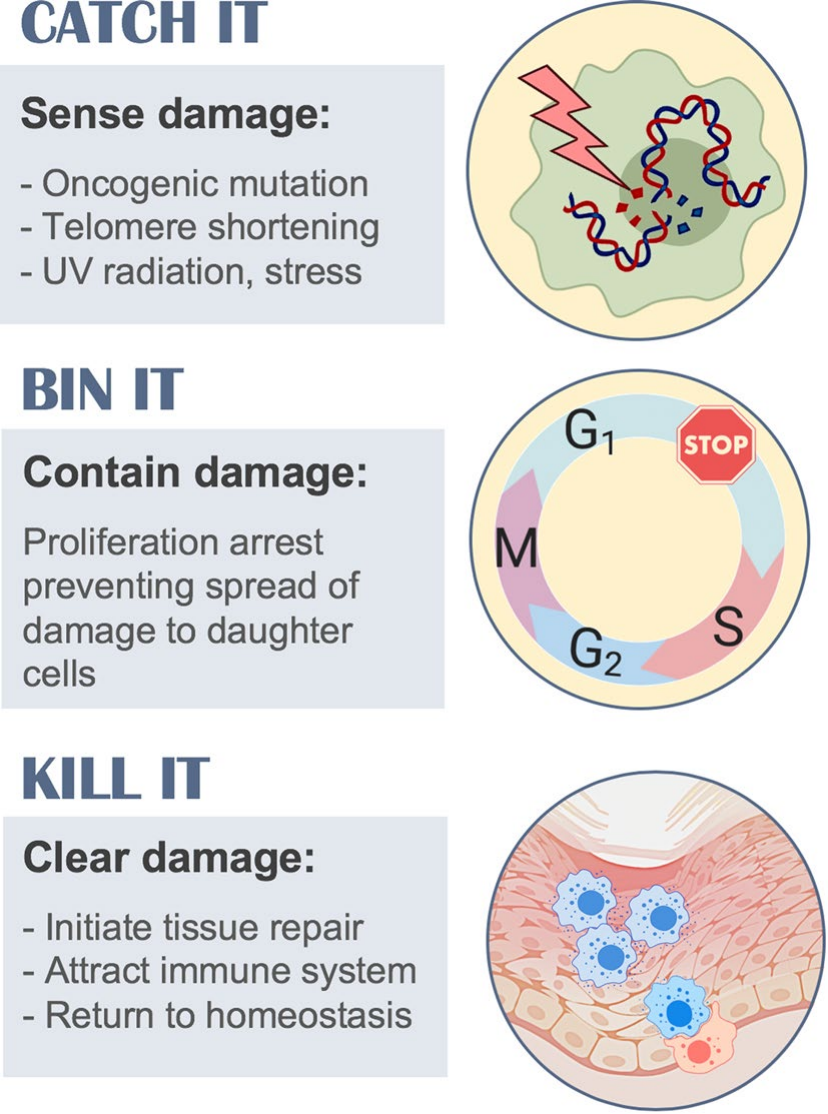
The purpose of senescence. Cellular senescence is initiated upon detection of cellular damage or stress. The damage is contained by preventing spread to daughter cells and finally cleared by eliminating damaged cells and inducing tissue repair

One of the major processes that drives resolution is the effective and timely elimination of apoptotic immune cells once they have completed the function for which they were recruited to the tissue. Consequently, the process of efferocytosis [48], or phagocytosis of apoptotic cells, is commonly used as a functional assay to assess the potential of new pro-resolving candidate molecules. Efferocytosis is crucial to achieve resolution because non-cleared apoptotic cells will turn necrotic, releasing damaging contents which further promote inflammation [48, 49] (Fig. 1). Similarly, senescence seems to follow a cycle of sensing a harmful stimulus followed by containment and elimination, a *catch-it-bin-it-kill-it* model (Fig. 2). For many years, it has been debated whether in certain circumstances, like in ageing, the process of senescence was per se detrimental, quite in line with the antagonistic pleiotropy model [6], suggesting the existence of late-acting harmful genes. There is now substantial evidence stating that senescence may always favour a protective role but it is the inefficiency of the immune system what reveals the dark face of senescence [50, 51]. An inefficient coupling of the immune system to complete the senescence programme and close the cycle allows the persistence of senescent cells in tissues for long periods of time while releasing pro-inflammatory factors that promote chronic inflammation and damage (Fig. 1). The reasons why this clearance may fail will be discussed in detail.

We propose here that the process of cellular senescence represents another endogenous protective pro-resolving mechanism that when dysregulated, it leads to persistent inflammation and sustained damage, and similarly to other pro-resolving mechanisms, it can be targeted to favour resolution. We recently demonstrated that the activation of the pro-resolving melanocortin receptor 1 (MC_1_) induced senescence in hyperactivated synovial fibroblasts from rheumatoid arthritis (RA) patients and favoured resolution of inflammatory arthritis in a serum-transfer mouse model [34]. RA is characterized by persistent inflammation in synovial joints, in which fibroblasts play a crucial role in the chronicity of the process [39]. Synovial fibroblasts present features of tumour-like cells including excessive proliferation and invasiveness of surrounding tissues. Although non-malignant, somatic mutations in tumour suppressor genes can be found, which have been shown to be epigenetically transformed into a permanent state of activation [52]. This imprinted aggressive behaviour not only sustains the pro-inflammatory microenvironment, but it is also responsible for the destruction of cartilage and bone within the joints. Thus, induction of senescence in these cells by activating the pro-resolving receptor MC_1_ can stop the cycle of reciprocal activation and favour resolution of inflammation [34].

This pro-resolving model of senescence provides a framework that solves the paradoxical co-existence of physiological and pathological consequences of senescence, and provides a much clearer view on why, when and how, senescent cells should be targeted. A failure to complete the programme that brings tissues back to homeostasis after a previous insult is what determines the detrimental effects of senescence, something largely determined by the context, as explained below.

## The bright and dark sides of senescence: reconciling opposite views

### Senescence in context

**Fig. 3:**
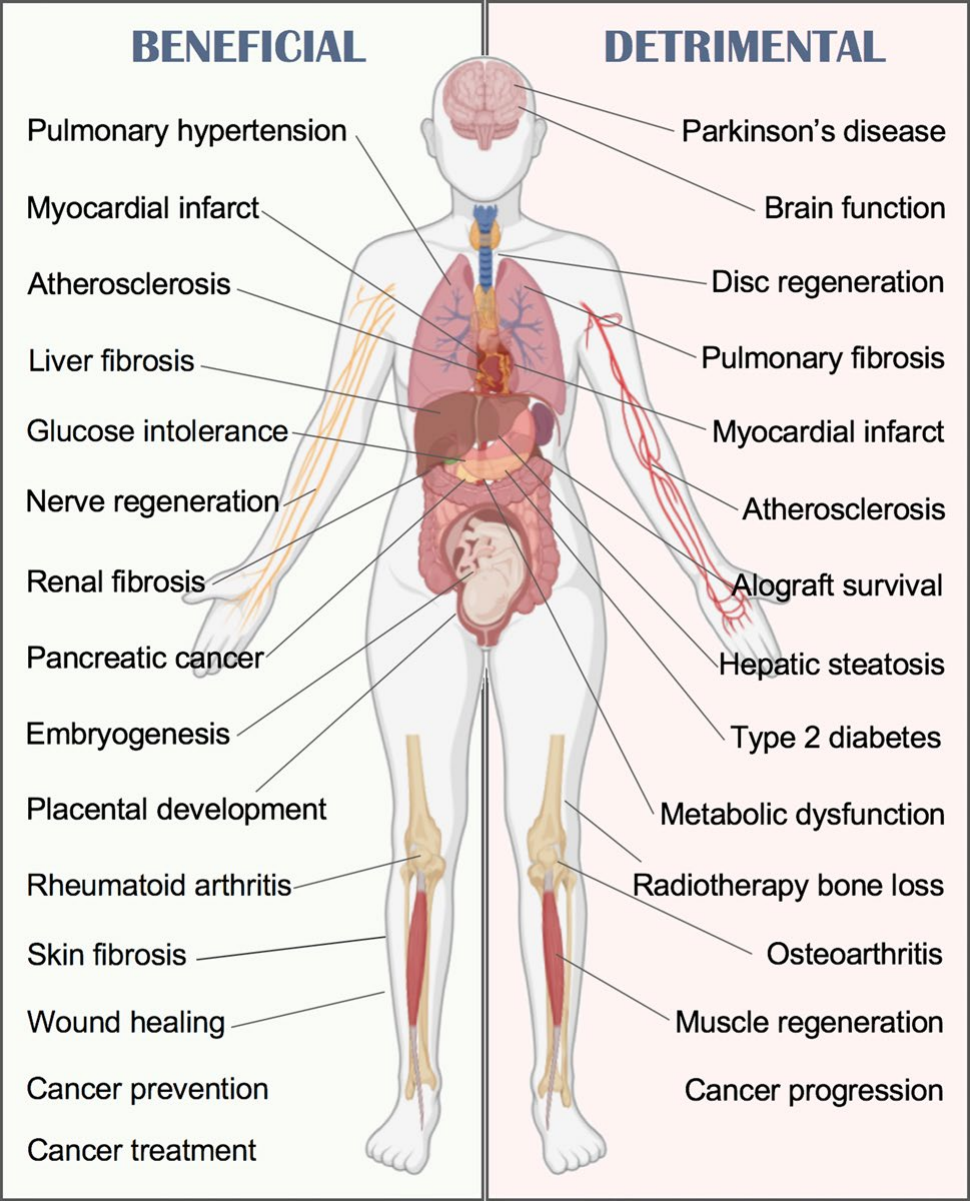
Physiological and pathological consequences of senescence. Beneficial and detrimental effects of senescence have been reported, suggesting the implementation of both pro- and anti-senescence approaches depending on the context

Scientific literature contains as much evidence for the protective actions of senescence as for its detrimental role (Fig. 3). In addition to cancer prevention and treatment, promotion of wound healing and embryogenesis, beneficial effects of senescence have been observed in liver [33, 53], renal [54] and skin [30] fibrosis, myocardial infarct [55], pulmonary hypertension [56], nerve regeneration [57], metabolic dysfunction[58], atherosclerosis, and rheumatoid arthritis [34, 59]. On the other hand, senescence may worsen intervertebral disc regeneration [60], fibrotic pulmonary disease [61], hepatic steatosis [62], Parkinson’s disease [63], allograft survival [63], muscle regeneration [64], brain function [65], osteoarthritis[66], type 2 diabetes [67] and radiotherapy-induced bone loss [68]. Paradoxically, detrimental effects of senescence have also been reported in myocardial infarct [69] and dysfunction [70], cancer progression [71], metabolic dysfunction [72] and atherosclerosis [73, 74].

**Fig. 4:**
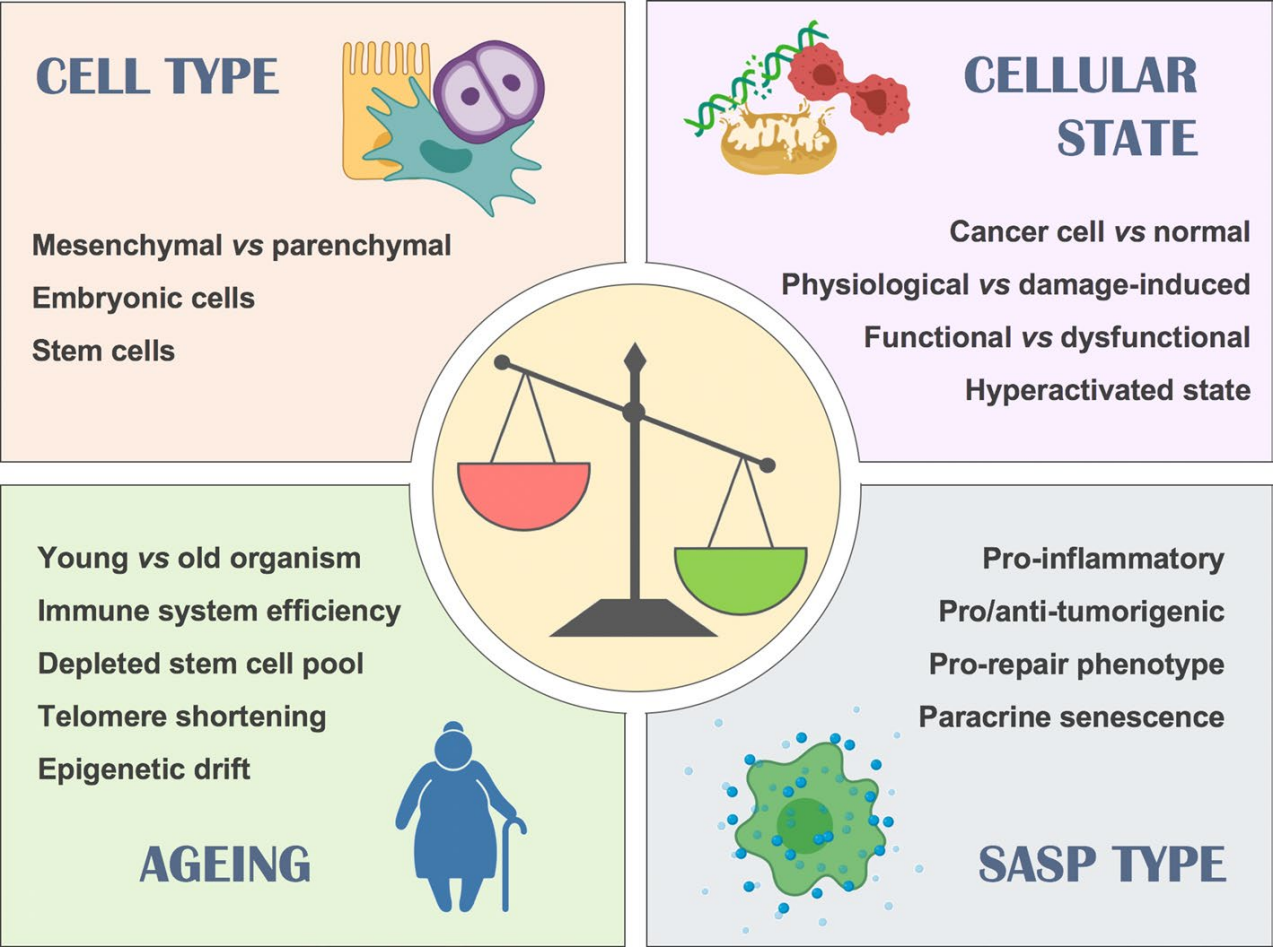
Contextual aspects determining senescence outcome. Whether the results of senescence are beneficial or pathological, this is largely determined by multiple aspects, commonly overlapping. The most important ones include: cell type, cell state, type of SASP released and age, which has a strong influence on the surveillance capacity of the immune system

However, evolutionary speaking, such a conserved mechanism would not have evolved if it were detrimental to the host. Amassing the existing literature on the subject, it is clear that multiple factors are involved in defining the impact of cellular senescence. We propose the consideration of four main contextual aspects (cell type, cellular state, SASP type and ageing) that largely determine the protective or adverse outcome of senescence (Fig. 4). First, the *cell type* affected by senescence can define the result. For example, senescence occurring in hepatocytes, the parenchymal cells found in the liver, results in dysregulated fat deposition, leading to hepatic steatosis [62]. However, when senescence is induced in myofibroblasts or in hepatic stellate cells, which are mesenchymal cells involved in healing by acquiring a fibroblast-like phenotype upon injury, the effect can be beneficial by limiting fibrosis [33, 53]. In myocardial infarct, senescence happening in the functional parenchymal cells of the heart, i.e. cardiomyocytes, increases mortality in mice [69], while if senescence occurs in cardiac fibroblasts, it promotes tissue repair [55].

The *cellular state* is another major determinant of the outcome of senescence. In rheumatoid arthritis, synovial fibroblasts are epigenetically locked in a hyperactivated state, sustaining immune cell recruitment and activation within the joints that further promotes inflammation and damage. This vicious cycle can be broken by inducing senescence in those fibroblasts, favouring the resolution of inflammation [34]. A different scenario takes place in osteoarthritis, in which senescence occurring in cartilage producing chondrocytes (parenchymal, indeed), is associated with cartilage degeneration [66].

Induction of senescence in a transformed cell is unequivocally beneficial to prevent cancer at an early stage. However, the *type of SASP -*third aspect of context*-* released in each particular case can greatly influence the outcome at later stages of tumorigenesis. Cancer cells can evade the immune system by various mechanisms and cancer immunotherapy is based on boosting the immune system to help find and/or destroy cancer cells. Then, the ability of the SASP to attract the immune system can contribute to the clearance of senescent tumour cells [75]. For example, CCL-5 secreted by senescent melanoma cells promotes the recruitment of tumour-infiltrating leukocytes leading to a tumour suppressive environment [76]. Conversely, in other forms of cancer, SASP contributes to cancer progression by the release of factors like IL-6 and IL-1α [71] as well as by other mechanisms including induction of epithelial–mesenchymal transition [77], mTOR activation[78], recruitment of immature myeloid cells [79], or induction of the checkpoint molecule PD-L1 [80]. Thus, the type of SASP plays an important role in favouring resolution and avoiding further tissue damage.

Beyond cancer, the frequent pro-inflammatory nature of the SASP is believed to be responsible for the toxic effects that accumulating senescent cells exert in aged organisms [81], which links to the fourth aspect of context, *ageing*. This was elegantly shown by Velarde et al. applying in young and old mice the model of mitochondrial dysfunction based on superoxide dismutase 2 (*Sod2)* deficiency, which leads to cellular senescence. They demonstrated that *Sod2* deficiency, and hence increased senescence, promotes wound healing and epidermal reepithelization in young mice, while the opposite was observed in old mice [82]. The pathogenic effect exerted by ageing on the outcome of senescence is largely determined by factors like stem cell exhaustion [83] and by the fitness of the immune system, as discussed next.

### The crucial role of the immune system

The completion of the senescent programme, the *kill-it* step (Fig. 2), requires the effective and timely cooperation of the immune system, and possibly, this is the most important factor for determining a successful resolution (Fig. 1). Immune surveillance of senescent cells is mainly carried out by natural killer (NK) cells, macrophages and T cells. NK cells target senescent cells via the receptor NKG2D binding to the ligands MICA and ULBP2 on the surface of senescent cells, and mediate their killing by the release of perforin and granzymes [84, 85]. CD8^+^ and CD4^+^ T cells are also involved in the elimination of senescent cells [86, 87]. The completion of the clearance to favour resolution requires the involvement of phagocytic cells like macrophages. Enrichment of F4/80^+^ cells was detected around areas of senescent cells in postpartum uterus, and their depletion caused enlargement of those areas [88]. In addition, it was found that monocytes/macrophages were required to complete the clearance of senescent hepatocytes after CD4^+^ T cells activity [86]. Macrophage plasticity may also be important and can be influenced by the SASP. Senescent stellate cells were shown to induce macrophage class-switch towards M1 phenotype favouring their clearance, while proliferating cells induced an M2 phenotype creating a tumour-promoting environment [89]. This co-existence of non-senescent cancer cells seems to be essential in the final outcome of senescence as it has also been shown that non-senescent hepatocellular carcinoma cells block the maturation of CCR2^+^ myeloid precursors preventing the clearance of senescent cells, favouring tumour growth [79].

The impact that organismal ageing exerts on the efficiency of the immune system and its surveillance capacity seems to drive the persistence of senescent cells within tissues and consequently their pathogenic effects. In a bleomycin model, it has been shown that senescent cell turnover can take from days in young mice to weeks in old animals [50], and that the reduced CD8^+^ T cell cytotoxic activity associated with age accelerates the accumulation of senescent cells [51]. However, inefficient clearance is not always due to an intrinsic defect in immune cells. For the immune system to find senescent cells, these have to be discoverable. Senescent cells can play ‘hide and seek’ and evade the immune system by the expression or release of certain mediators. The mechanisms for clearance of apoptotic cells are well understood and require the release of *find-me* signals (e.g. S1P, CX3CL1), with a balanced expression of *eat-me* (e.g. phosphatidylserine, calreticulin) and *do not-eat*-me signals (e.g. CD47, CD31, PAI-1) [90, 91]. These signals have been extensively investigated and exploited to promote resolution of inflammation [92–94]. Although these signals are not so well understood for senescent cells, several do not-eat-me signals have been reported, like expression of checkpoint molecule HLA-E, which blocks NKG2D-mediated cytotoxic response by NK and CD8^+^ T cells [87], or expression of decoy receptor 2, Dcr2, which inhibits the activation of death receptors 4 and 5 [85]. Other mechanisms may exist that render senescent cells immunologically cold and appropriate immune recognition signals are necessary to generate a response by immune cells. Moreover, the expression of these signals may depend on the influence of context-specific microenvironments. On the other hand, post-translationally modified vimentin has been proposed as an eat-me signal for senescent cells [95]. Little is known about whether additional eat-me signals exist in senescent cells, or if the senescent programme evolved to be coupled to subsequent apoptosis, relying then on the typical apoptosis eat-me signals to complete clearance.

## Not all senescent cells are created equal

To produce a unified classification of senescence has proven difficult as multiple criteria could be applied: replicative or premature, presence or absence of DNA damage, intrinsically or extrinsically induced, damage-induced or developmentally programmed, and more. It is striking how a rather simple phenomenon, cell cycle arrest, can generate a vast diversity of cellular states. Multiple stimuli can result in senescence via multiple mechanisms, in multiple cell types, resulting in multiple SASP types, and multiple outcomes. Senescence heterogeneity occurs at many levels with implications in disease phenotypes, potential therapeutic approaches, in the way we conduct research and in the interpretation of results.

Several and diverse types of stimuli can induce senescence, including telomere attrition [5], oncogenic transformation [21], PTEN loss [96], mitochondrial dysfunction [97], cytosolic DNA [98], chemicals and genotoxic drugs [99, 100], disturbed blood flow [101], ultraviolet radiation [102], cell fusion [103], proteasome inhibition [104], or by melanocortin receptor 1 agonism [34]. The mechanisms by which senescence is executed also differ. While p16 (*CDKN2A*) plays a prominent role in oncogene-induced senescence [21], it is mainly p21 (*CDKN1A*) that mediates senescence during telomere loss [105] and embryonic development [11]. In hepatic tumour cells, however, it was shown that cells can evade senescence even when p16, p19, p21 and p53 are all expressed [106]. Moreover, the pro-senescence effect of p16 in human cells is more prominent than in mouse cells [107]. The DNA damage response mediates senescence as a result of telomere loss [105, 108] and in certain types of oncogene senescence [3], but not when senescence is elicited by mitochondrial dysfunction [97] or by developmental cues [11].

Heterogeneity in the SASP composition is also well documented. Although in general terms, the SASP is typically described as pro-inflammatory, there are many examples in which this is not the case. Senescence induced by mitochondrial dysfunction in human fibroblasts lacks IL-1-related cytokines [97], and senescence induced by ectopic expression of p16 does not increase typical SASP cytokines like IL-6, IL-8 or CXCL-1 [109]. Senescence induced by proteasome inhibition in lung fibroblasts also leads to a predominantly non-inflammatory secretome [110]. In addition, atypical non-inflammatory SASP is also associated with senescence induction by certain drugs, like treatment with doxorubicin on endothelial cells [111] and MC_1_-selective ligands on synovial fibroblasts [34]. It has been suggested that persistent DNA damage mediates cytokine secretion by senescent cells [112] via the activation of NF-κB [113]. It might then be plausible that in those types of senescence processes lacking a DDR response, the resulting SASP will not be pro-inflammatory. It remains to be known which scenario will be more favourable to achieve resolution: a pro-inflammatory SASP able to call the immune system to induce clearance, or a silent SASP that does not cause damage to the surrounding tissues but may delay clearance.

The sensitivity of cells to senolytics is also heterogeneous. Senolytics are drugs that induce lysis of senescent cells and are under investigation as potential therapies for cancer, ageing and other conditions. However, depending on the type of senescence, some senolytics may be more effective than others. The senolytic Bcl-2 inhibitor ABT-263, navitoclax, was effective in inducing apoptosis in murine embryonic fibroblasts [114], but ineffective in murine sarcoma cells overexpressing p16 [115]. Navitoclax was also ineffective in inducing cell death in melanoma cells, an effect attributed to increased levels of anti-apoptotic MCL-1. Interestingly, co-administration with the MCL-1 inhibitor S63845 markedly sensitized the cells to death by navitoclax [116].

**Fig. 5:**
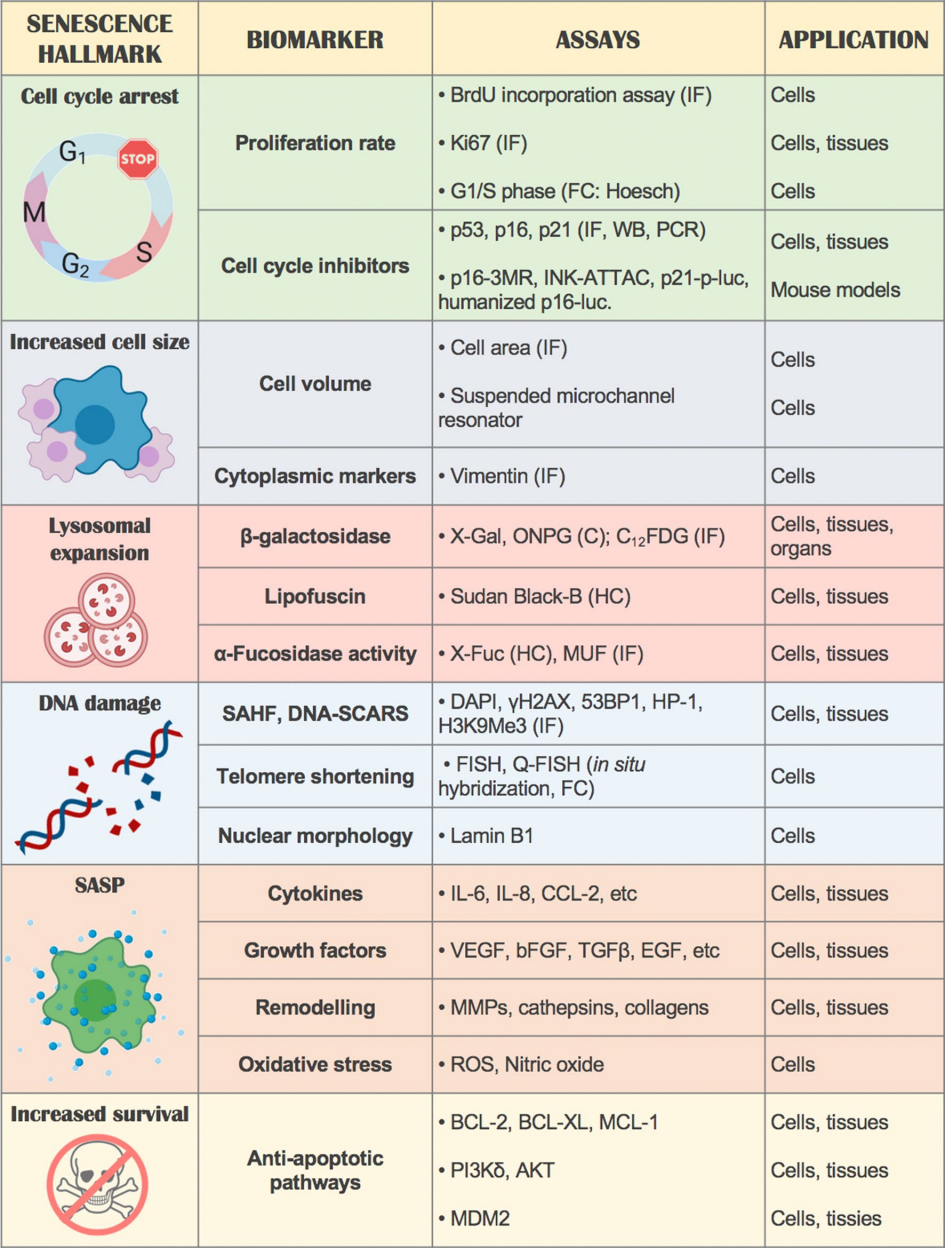
Hallmarks of senescence and biomarkers used in research. Cell cycle arrest ([122, 166]) increased cell size ([10, 166, 177]), lysosomal compartment expansion ([178–180]), DNA damage ([117, 181–184]), SASP components ([4]), anti-apoptotic pathways ([185]). Abbreviations: Immunofluorescence (IF), flow cytometry (FC), western blot (WB), colorimetric (C), histochemistry (HC), DNA damage response (DDR)

One of the major implications of this multi-level heterogeneity is the lack of universal markers of senescence, which impacts scientific research in particular when comparing different studies. Multiple markers have been described, some of which are more common than others among the different types of senescence and are typically used in senescence research (Fig. 5). The most frequently used include the overexpression of cyclin inhibitors, detection of β-galactosidase reflecting lysosomal expansion, decrease of proliferation rate, detection of heterochromatin foci or markers of DNA damage and release of pro-inflammatory cytokines. However, as previously discussed, these features are not always present. Other markers have also been described in specific types of senescence like lamin B1 loss in HCA2 fibroblasts [117], MGST1 in COPD-derived fibroblasts [118], β3 integrin in primary fibroblasts [119] and WNT16B during replicative senescence in fibroblasts [120]. If we accept the definition of cellular senescence as cell cycle arrest with maintained cellular growth, as a minimum, measurements or markers indicative of decreased proliferation (cell number, proliferation marker Ki67, cell cycle phases, etc.), accompanied with markers indicative of increased growth or metabolic activity that distinguishes senescence from other cell arrested cellular states (e.g. increased cell size, lysosomal expansion reflected in β-galactosidase or lipofuscin content, etc.) should be used to determine the presence of senescence (Fig. 5). Other common hallmarks of senescence, however, may require the analysis of specific biomarkers, like particular SASP components [4, 113, 121], presence of DNA damage or involvement of specific CDK inhibitors. This may help to define the specific type of senescence as they tend to be context-dependent. This approach, while feasible during in vitro studies, presents difficulties in detecting senescence in vivo. Typically, detection of cell cycle inhibitors, β-galactosidase in tissue sections or whole organs, lipofuscin staining or reporter mice like the p16-3MR [32], INK-ATAAC [122], humanised p16-luc [123] and p21-p-luc mice [124] are currently available.

As universal markers may not exist, the creation of a catalogue compiling all types of senescence, with features and markers characterizing each one, would represent a very helpful initiative. Such a resource might include sections like cell type affected, physiological/pathological context, inducing stimulus, DNA damage involvement, participating cyclin inhibitors or SASP components. Recently, the literature-based resource SeneQuest (https://senequest.net) have been made available to search for genes related to senescence.

## Senescence in the pipeline

### Opportunities for therapeutic exploitation of senescence

**Fig. 6:**
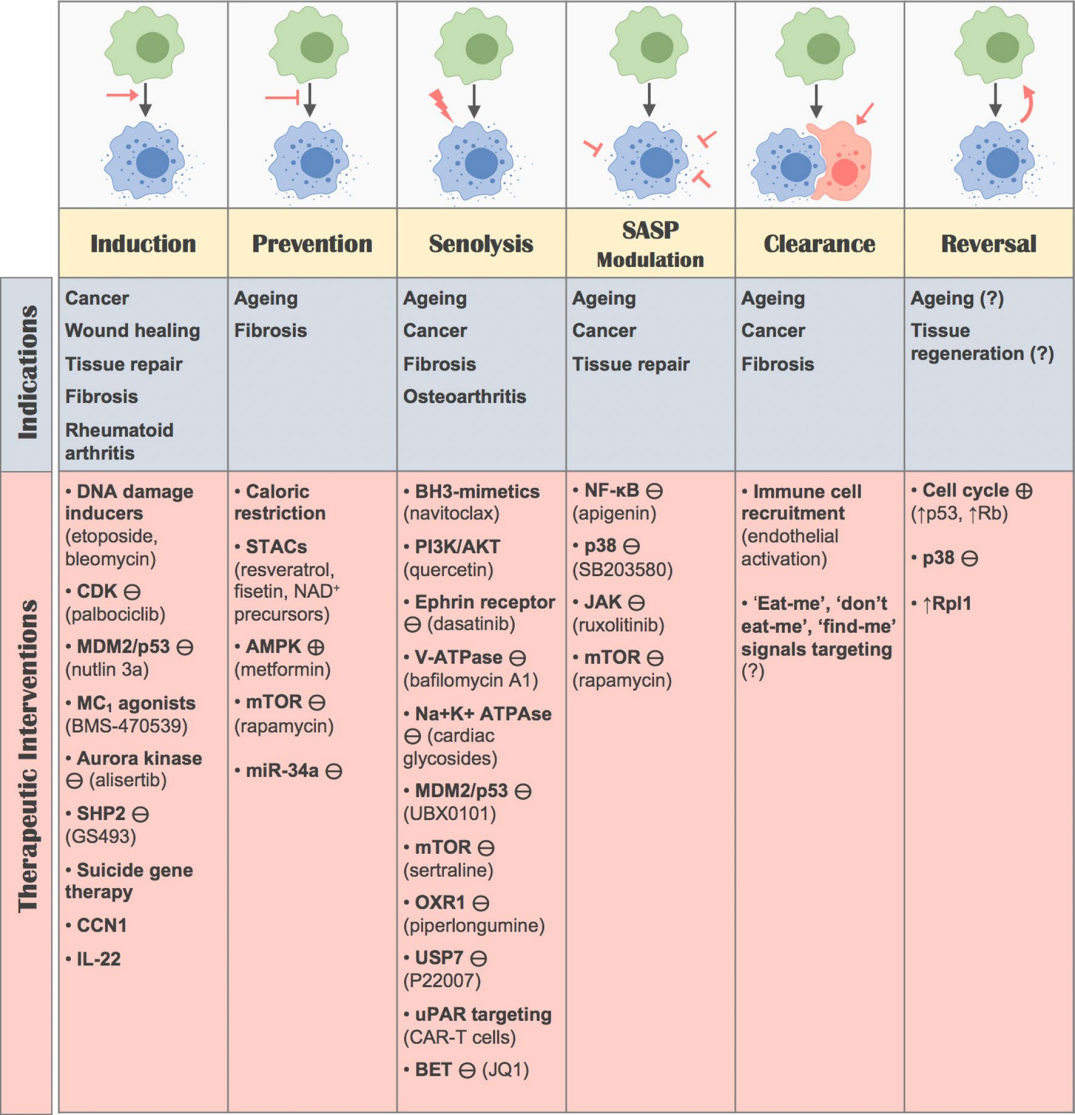
Potential therapeutic strategies based on senescence. Senescence can be targeted from multiple angles and has the potential to benefit multiple therapeutic indications. Abbreviations: activator/agonist ( ⊕), inhibitor/antagonist ( ⊖), overexpression (↑)

Cellular senescence offers an arsenal of potential therapeutic uses: for some, its intrinsic anti-cancer properties can be further exploited, while for others, senescence holds the secrets of youth. However, the bi-directional nature of these options, that is promotion or elimination depending on the target disease, presents important challenges: what is beneficial for one purpose may potentially be detrimental for another. The therapeutic potential of promoting, preventing, killing, modulating and clearing senescence will now be discussed (Fig. 6).

Several pro-senescence drugs are currently approved for the treatment of cancer, including methotrexate, etoposide, tamoxifen, palbociclib, doxorubicin or cisplatin, among many others, and generally induce senescence by a DNA-damage-mediated mechanism or interference with cell cycle components, among other mechanisms [99, 100, 125, 126]. Side effects derived from persistent SASP or from the pro-senescence action in off-target tissues limit their use [126, 127]. However, the induction of senescence also represents an opportunity to generate new vulnerabilities in cancer cells. Wang et al. demonstrated that p53 mutant liver cancer cells made senescent by treatment with CDC7 kinase inhibitors, become susceptible to senolysis by the antidepressant sertraline [128]. Interestingly, it has also been reported that p53 mutant breast cancers are resistant to chemotherapy with doxorubicin when co-treatment with hormone therapy is not used, because cells bypass apoptosis and become senescent. However, these p53 mutant cells rendered senescent by doxorubicin became sensitive to tamoxifen [129]. Hence, this double-hit approach suggests that induction of senescence followed by senolysis can enhance therapeutic efficacy in cancer. Induction of senescence to favour wound healing and induce resolution of fibrosis or chronic inflammatory conditions like rheumatoid arthritis may require less toxic approaches like the recently discovered pro-senescence activity of MC_1_-selective agonists [34], acting via the activation of a highly druggable GPCR which ligands have been shown to be safe in clinical trials. Other pro-senescence approaches reported include the inhibition of aurora kinases [130], Shp2 inhibitors [131], suicide gene therapy [115], MDM2/p53 interaction inhibitors [132], treatment with CCN1 protein [30] or interleukin 22 [133]. Interestingly, it was also shown that reprogramming approaches by ectopic expression of OSKM or Yamanaka factors (*OCT4*, *SOX2*, *KLF4*, *MYC*) to induce pluripotent stem cells (iPS) is strongly associated with induction of senescence where senescent cells create a tissue context involving IL-6 that favours OSKM-driven reprogramming in surrounding cells [134].

Complete suppression of senescence will invariably promote cancer development. However, there might be ways to prevent certain types of stress-related senescence by reducing the relevant stress signals. This approach is based on the information theory of ageing, suggesting that the accumulation of damage at the cellular level decreases cellular repair mechanisms, leading to decreased functionality of the whole organism. Ageing produces accumulation of DNA instability leading to re-distribution of the epigenetic regulators sirtuins to engage into repair mechanisms as a survival circuit, which is accompanied by disengagement of other cellular activities. This epigenetic noise (or information loss) caused by exhaustion of the survival mechanism, leads to loss of cellular identity terminating in senescence. Although this theory, so far, has only been fully demonstrated in yeast [135, 136], substantial evidence suggests a mayor role of sirtuins in eukaryotic cells senescence too. Sirt1 prevents telomere shortening in mice [137], while Sirt6 overexpression prevented replicative senescence in human cells [138]. Caloric restriction, by inducing sirtuins and enhancing DNA repair mechanisms, can reduce senescence burden in ageing and extend lifespan [139]. Caloric restriction can be mimicked with sirtuin-activating compounds (STACs) such as resveratrol, fisetin, or NAD^+^ precursors, which extend the lifespans of yeast, mice and humans [140–142]. Consistently, it has been shown that the inhibition of Sirt1 promotes premature senescence in mouse fibroblasts [143]. However, sirtuin inhibitors like sirtinol [144] present anti-senescence activity, suggesting that further research is needed to fully elucidate the role of sirtuins, or their targeting, in senescence. Indeed, it has been suggested that the dual outcome of sirtuin inhibition might depend on p53 status [145]. STACs act by activating AMPK which in turn inhibits mTOR. Hence, AMPK activators like metformin and mTOR inhibitors like rapamycin also prevent senescence and ageing [142, 146]. Inhibition of SIRT1 was also achieved by expression of the microRNA miR-34a, reducing senescence in human adipose tissue-derived mesenchymal stem cells [147].

The elimination of senescent cells using senolytics is being intensively investigated for applications in cancer, ageing and facilitation of resolution in fibrosis. Hence, under the right context, senolytics may also act as pro-resolving drugs. Senolytics aim to induce death in senescent cells without affecting proliferating or quiescent cells, by targeting a wide range of vulnerabilities existing specifically in senescent cells. These include the increased expression of BCL anti-apoptotic proteins (navitoclax and other BH3-mimetics), the PI3K/AKT pathway (quercetin), ephrin receptors (dasatinib), lysosomal V-ATPase (bafilomycin A1, concanamycin A), Na + /K + ATPase (cardiac glycosides), MDM2/p53 interaction (UBX0101), mTOR pathway (sertraline), oxidation resistance 1 (piperlongumine), USP7 protease (P22007), urokinase-type plasminogen activator receptor (CAR-T cells) and bromodomain and extra-terminal (BET) family proteins [66, 128, 148–154]. General cytotoxic drugs like the antibiotic duocarmycin can also be converted into senolytics by addition of a galactose motif, creating a pro-drug that is preferentially cleaved under high β-galactosidase expression, producing a targeted-delivery system for senescent cells [155]. Other antibiotics like azithromycin and roxithromycin have shown preferential cytotoxic activity in senescent fibroblasts [156], although the exact mechanism has not been reported. As previously discussed, different types of senescence display different sensitivities to senolytics.

A fourth type of approach to targeting senescence consists of using drugs that modulate the composition of the SASP, commonly termed senomorphics. The search for senomorphics has been focused on the discovery of drugs that reduce the pro-inflammatory activity of the SASP. For example, NF-κB inhibitors like apigenin or glucocorticoids can reduce the pro-inflammatory nature of the SASP [157, 158], as well as p38 inhibitors and JAK inhibitors, like SB203580 molecule and ruxolitinib, respectively [159, 160]. The natural compound rapamycin, via inhibition of mTOR, also modulates the composition of the SASP towards a less damaging secretome, and has shown to increase lifespan in mice [161, 162]. A senomorphic therapy is aimed at reducing the detrimental effects of a persistent SASP without the need of eliminating the senescent cells, which might be more advantageous than a senolytic as it may allow senescent cells to perform their physiological and reparative actions, without fuelling further inflammatory processes; however, this view needs to be corroborated. Therapeutic areas for potential application of senomorphics include the prevention of ageing associated pathologies, reduction of inflammatory pro-tumour microenvironment in cancer and reduction of damaging SASP during fibrosis.

Clearance of senescent cells can also be promoted therapeutically to facilitate their removal from tissues. The pro-tumorigenic effect of senescent cells derives from their persistence in tissues after containing the oncogenic damage. Thus, strategies that promote the surveillance capacity of immune cells may contribute to tumour regression. Ruscetti et al. demonstrated that treatment with palbociclib induced senescence in pancreatic cancer with a pro-angiogenic SASP, which favoured the recruitment of CD8^+^ T cells. These recruited cells showed signs of exhaustion but the combination of pro-senescence therapy with blockade of PD-1 checkpoint triggered T cell anti-tumour activity [19]. Hence, therapies to enhance clearance by immune system require not only the recruitment of appropriate cells, but also to ensure that their cytotoxic activity is intact. In addition, as discussed above, senescent cells need to be visible to the immune system to trigger their phagocytosis. Enhancement of apoptotic cell clearance can be achieved for example, by targeting the eat-me signal phosphatidylserine with a modified annexin A5 that interacts with αvβ3 receptors on the surface of macrophages [92]. This approach not only favoured resolution but it also induced the release of anti-inflammatory IL-10 by the phagocyte. This switch from pro- to anti-inflammatory signals during efferocytosis has been referred to as “tolerate me” signals [163]. Whether a similar non-phlogistic mechanism occurs during senescent cell engulfment remains to be studied. Another example of efferocytosis enhancement includes the matricellular protein CCN1, which facilitates the clearance of apoptotic neutrophils by a similar mechanism, bridging the apoptotic cell with the phagocyte [164]. Interestingly, as mentioned earlier, CCN1 induces senescence during physiological wound healing but whether CCN1 could also act as an opsonin for senescent cells has not been determined. On the other hand, blocking the do not-eat-me signal CD47 was demonstrated effective in promoting phagocytosis of apoptotic cells and preventing atherosclerosis [165]. The identification of similar signals that selectively tag senescent cells may lead to similar pro-phagocytic approaches as the one already existing for apoptosis.

Finally, reversal of senescence is typically investigated with the aim to discover factors that mediate the irreversibility of senescence [166]. It was found that telomere-induced senescence may be reversed by p53 or Rb inactivation if p16 is expressed at low levels [167], indicating the requirement of high levels of p16 to sustain the irreversible state. Non-replicative senescence in CD4^+^ memory T cells showed dependence on the p38 pathway [168]. Hypothetically, the reversal of senescence may benefit ageing-related pathologies by enhancing immunosenescence or by replenishing depleted stem cell pools. However, this approach brings the risk to spread the damage that led to senescence induction in the first place. The protein Rpl1 induces the bypass of replicative senescence but also contributed to transformation in ras^Val12^ mutant cells [169]. It is also believed that the transformation from nevi (senescent melanocytes) to melanoma occurs when senescent cells regain the ability to proliferate by acquiring additional abnormalities, hence reverting their senescent status [170].

### Ongoing approaches in clinical development

**Fig. 7:**
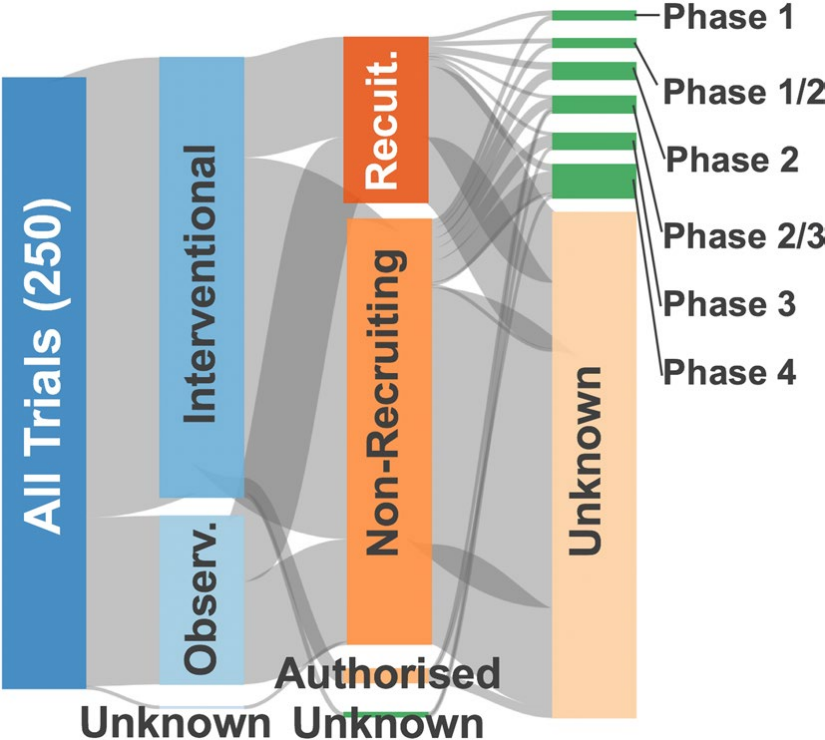
Clinical trials related to senescence. Information retrieved from the WHO International Clinical Trial Registry Platform in July 2020 using the search terms: senescence, senescent, senolytics, senolysis, senomorphic, senotherapy, senotherapeutic. This figure shows the classification of the trials according to different criteria: observational/interventional, whether they are actively recruiting or not, and the trial phase. Of the 180 interventional studies identified, 41 were actively recruiting. Of the 69 observational studies, 27 were actively recruiting. A selection is shown in Table 1

Despite all the existing challenges, multiple clinical investigations with senescence-related approaches are currently undergoing. We identified 250 clinical trials registered at the World Health Organization repository with the majority (180) being interventional (Fig. 7, Table 1).

**Table 1:** Clinical trials investigating senescence-related therapies

Trial ID	Condition	Intervention	Sponsor
NCT02102724	HIV-Related Immune Senescence	Fish oil omega-3	Rush University
NCT03100799	Biomarkers of Senescence in Osteoarthritis of the Knee	Arthroscopy, Arthrocentesis, MRI	Unity Biotechnology, Inc
NCT03338985	Role of Senescence in of Endometrial Cancer	Genetic analysis	CHU de Reims
NCT04113122	Senescence After Chemotherapy for Testicular Cancer	Skin, fat biopsy	University Medical Center Groningen
NCT03513016 *Phase I*	Osteoarthritis of the Knee	UBX0101	Unity Biotechnology, Inc
NCT03353597 *Phase I/II*	Reversing Epigenetic & Senescence by Transfusing Young Plasma	Plasma Transfusion	Chandra Duggirala
NCT04063124 *Phase I/II*	Progression of Alzheimer's Disease	Dasatinib, Quercetin	University of Texas
NCT04210986 *Phase I/II*	Osteoarthritis Cartilage Degeneration	Fisetin	Steadman Philippon Research Institute
NCT02533180 *Phase II*	Donor Specific Immune Senescence and Exhaustion—Liver Transplantation	Immunosuppression withdrawal	NI of Allergy and Infectious Diseases
NCT04313634 *Phase II*	Skeletal Health in Older Humans	Dasatinib, Quercetin, Fisetin	Sundeep Khosla
NCT02848131 *Phase II*	Chronic Kidney Disease	Dasatinib, Quercetin	Mayo Clinic
NCT00461695 *Phase IV*	Persistent CMV infection on Immune Senescence	Vaccination against TBEV	University of Zurich
NCT01827462 *Phase IV*	Immune Responses—Influenza Vaccine	Inactivated vaccine	Stanford University
EUCTR2012-003,987–34-BE	Senescence markers—Kidney Transplantation	Metformin	UZ Leuven
KCT0003477 *Phase III*	Senescence in type 2 diabetes with Hypercholesterolemia	Rosuvastatin, ezetimibe	Chungnam National University Hospital
NCT02368821	Senescence in Placenta from Complicated Pregnancies	Observational	Meir Medical Center
JPRN-UMIN000016565	Cellular Senescence in COPD	Observational	Tohoku University
JPRN-UMIN000040058	Senescence cell in Diabetic Hand Syndrome	Observational	Nagoya University,
NCT02755584	Senescence During Wound Healing	Observational	National Institute on Aging
NCT03559569	Senescence in Circulatory Shock	Observational	University Hospital, Strasbourg
ChiCTR2000031492	Osteoarthritis After Anterior Cruciate Ligament Tear	Observational	Shanghai Jiaotong University

Information retrieved from the WHO International Clinical Trial Registry Platform on July 2020 using the search terms: senescence, senescent, senolytics, senolysis, senomorphic, senotherapy, senotherapeutic. A selection from 250 trials identified is shown

Unity Biotechnology Inc. has completed a phase I placebo-controlled trial where the primary outcome was to address the safety and tolerability of a single intra-articular injection of UBX0101, monitoring the incidence of serious and non-serious adverse events in knee osteoarthritis (NCT03513016). The compound is reported by the company as an inhibitor of the interaction between MDM2/p53, and has displayed senolytic properties in pre-clinical research [171]. A phase II trial with ~ 180 participants is currently ongoing to investigate the efficacy of a single dose on osteoarthritis pain score (NCT04129944). A small pilot study in five participants with Alzheimer’s disease is being conducted to assess brain penetrance of the senolytic compounds dasatinib and quercetin (NCT04063124). Another trial has investigated the efficacy of these two senolytics, given orally, in eliminating senescent cells in chronic kidney disease patients (NCT02848131). Results for this trial, led by Mayo Clinic, have been published [171], reporting significant decreases in senescent cell burden. Oral administration of fisetin, a sirtuin activator but also reported as senolytic, is being trialled in ~ 70 patients with osteoarthritis to address safety (NCT04210986). Observational studies on the role of senescence in several diseases are also being conducted, including COPD, diabetes, wound healing, complicated pregnancies and circulatory shock (Table 1).

### Challenges in senescence research and potential therapies

While reviewing the recent advances in senescence research and directions towards potential clinical applications, we identified a number of challenges, gaps and research paths that may help advance the field of senescence and accelerate its successful translation into clinics.

We now know that senescence comes in many flavours, induced by multiple mechanisms leading to different SASPs, that may, or may not, be pro-inflammatory. Senescence is intensively studied in the context of fibrosis where bleomycin is used to induce the disease in animal models. However, bleomycin itself induces senescence by causing direct DNA strand breaks [172], suggesting that the SASP and its actions may greatly differ from the ones produced by endogenously induced senescence during would healing and fibrosis, and as such, results may not be extrapolatable into human disease. Fibrosis models have also been used to test senolytics and generally, when such treatments improve fibrosis, it is believed that senescent cells were the pathogenic cells. However, as Lagares et al. demonstrated, senolytics can also target and kill activated (i.e. non-senescent) myofibroblasts [47], which are major drivers of fibrosis. Therefore, the protective effects of senolytics in fibrosis should not be directly attributed to the elimination of senescent cells without performing more in-depth investigations.

Pro-senescence therapies are currently used to treat several forms of cancer. The major obstacles derive from the induction of senescence in off-target tissues, which may promote their premature ageing. Hence, selective and targeted senescence to cancer cells would be preferred. The group of Bernards et al. is working in that direction by performing screenings of pro-senescence targets in cancer cells. They identified not only molecules that induce senescence specifically in liver cancer cells, but, following a second screening, they also found specific senolytics which favour their elimination [128]. This is an excellent example of the integration of a pro-senescence followed by senolytic approach to ensure completion of the senescence programme, which they call the ‘one-two punch’ approach. Certain types of cancer are actually derived from mutations in genes related to senescence, like *CDKN2A*, inducing malignancy due to their ability to bypass senescence [24]. A better understanding of alternative routes to induce senescence may help to design novel therapies for those cancers.

Anti-senescence approaches are based on preventing the build-up of informational noise caused by cellular stress that leads to senescence. It has been reported that inhibitors of mTOR like rapamycin have anti-senescence, senolytic and senomorphic activity with potential to reduce age-related pathologies. However, rapamycin may also prevent oncogene-induced senescence by preventing up-regulation of p53, hence impairing our major endogenous anti-cancer mechanism from acting [96]. These potential detrimental actions require further investigation to ensure the safety of such approaches.

Some strategies focus on identifying common targets of senescent cells to develop broad-spectrum senolytics [149, 154, 155]. These can be very useful as research tools and will allow the comparison of different studies if all senescent cells, regardless of their type and context, respond to the same senolytic. However, selective senolytics for each type of senescence, and possible each cell type, may be more beneficial for therapeutic applications than a one-size-fits-all approach. For example, we may need to favour the elimination of senescent cells from a tumour while preserving senescent cells physiologically dealing with wound healing. This may reduce the therapeutic indications available for such drugs, but might provide a more balanced risk/benefit profile. However, this will require the identification of specific vulnerabilities for each type of senescence. In addition, all animal studies with senolytics, as with all studies essentially, are performed under controlled conditions where mice do not suffer from additional co-morbidities, traumas, or wounds. It is then yet unknown how senolytics will perform in more real-life situations. Another challenge related to senolytics is that the elimination of senescent cells may not restore organ or tissue function if not coupled with pro-regeneration approaches [45]. The elimination of senescent chondrocytes in osteoarthritis may help to reduce the sustained release of pro-inflammatory factors, but that alone may not lead to restoration of the chondrocyte population due to the intrinsic limited regenerative capability of this avascularised tissue. The same concept applies to fibrosis, where repopulation with functional parenchymal cells, not only elimination of senescent ones, is required for restoration of tissue function. The effects of senolysis on stem cell exhausted tissues may not restore function.

A further challenge is that senolytics are developed under the assumption that the immune system may recognise *dead* senescent cells, i.e. apoptotic cells. Speculating as we write, it may be possible that in certain contexts, phagocytes might not recognize senescent or apoptotic cells, rendering senolysis approaches detrimental, as deficient efferocytosis also leads to secondary cell necrosis, which would sustain pro-inflammatory damage and comport failed resolution. Indeed, inefficient efferocytosis is associated with pathologies like systemic lupus erythematosus due to genetic or acquired C1q deficiency [173]. How senolysis will perform in immunosuppressed patients also remains unknown. An interesting aspect of the clearance of senescent cells is that they are eaten alive, a process termed *phagoptosis* [174]. This may represent an opportunity for the development of strategies aimed at actively modulating the expression of surface molecules that target them for clearance, similar to approaches already under investigation that actively modulate the composition of the SASP. This is possible, because it is known that the SASP can be modulated without affecting the irreversibility of cell cycle arrest, as they seem to be controlled by independent regulatory networks. Cell cycle arrest is largely dependent on p16/Rb or p53/p21 pathways, whilst the SASP is more dependent on NF-κB, C/EBPβ or mTOR pathways [175] and inhibiting the latter ones does not revert cell cycle arrest.

The field of ageing research is also facing the difficulty in running the long clinical trials that will be required to assess if senolytics may indeed prevent age-related diseases. To facilitate research, the inclusion of organismal senescence (essentially ageing) as a disease category in the WHO International Classification of Diseases (ICD) has been proposed [176]: a major challenge, because accepting that ageing is a disease will have important social, political and medical implications. In the latest ICD version (ICD-11, https://www.icd.who.int), ‘old age’, under the code MG2A, is recognised as a *symptom, sign or clinical finding, not elsewhere classified*. Although age as a disease is difficult to accept by many, others are happy to conduct self-experimentation by taking available senolytics and STACs like quercetin or resveratrol. This kind of citizen science can be a double-edged sword. In the field of melanocortin research, due to self-administration of illegal melanocortin peptides to boost tanning, the development of melanoma on some of these individuals, likely derived from their risky behaviour in their sun exposure, led to the wrong suspicion that melanocortins may cause melanoma, a conclusion disproved in controlled clinical trials.

## Summary

Senescence has emerged in recent years as the rising star among the cellular processes to target to develop innovative therapies due to their potential impact on diverse therapeutic areas, such as cancer, fibrosis and ageing. We propose here the process of cellular senescence as an endogenous pro-resolving mechanism. If we consider their clearance as an integral step of the senescence programme, failure to complete this last step is what results in non-resolving inflammation. Furthermore, we reason on how senescence is a highly heterogeneous phenomenon and we suggest that a precise definition of context will benefit our understanding and enable reliable and meaningful comparison across different studies. Senescence can be targeted from multiples angles (promotion, prevention, killing, modulation, clearance) and it is important to consider the context to determine what, when and how we should target senescence. Whether beneficial or detrimental, this cannot be decided without taking the context into account.

Senescence passes the appraisal, with the set objectives of (i) improving detection strategies, (ii) consideration of contextual aspects to decide beneficial/detrimental role, (iii) understanding better how the process of clearing occurs and could be modulated, (iv) creation of repositories compiling features of all types of senescence (cell type, context, pathways, SASP, biomarkers), and (v) development of more targeted approaches for specific pathological situations. Neither zombie cells nor regenerative powers, deciphering the process of senescence has a great potential to improve human health, potential reflected in the prominent presence of senescence approaches currently under clinical development.

## Data Availability

Not applicable.
